# Relationship between functional disability and costs one and two years post stroke

**DOI:** 10.1371/journal.pone.0174861

**Published:** 2017-04-06

**Authors:** Ingrid Lekander, Carl Willers, Mia von Euler, Mikael Lilja, Katharina S. Sunnerhagen, Hélène Pessah-Rasmussen, Fredrik Borgström

**Affiliations:** 1 Ivbar Institute AB, Stockholm, Sweden; 2 Medical Management Center, LIME, Karolinska Institutet, Stockholm, Sweden; 3 Department of Clinical Science and Education, Södersjukhuset, Karolinska Institutet, Stockholm, Sweden; 4 Karolinska Institutet Stroke research Network at Södersjukhuset, Stockholm, Sweden; 5 Department of Public Health and Clinical Medicine, Unit of Research, Education, and Development, Östersund Hospital, Umeå University, Östersund, Sweden; 6 Institute of Neuroscience and Physiology, Rehabilitation medicine, University of Gothenburg, Gothenburg, Sweden; 7 Department of Health Sciences, Lund University, Lund, Sweden; 8 Department of Neurology and Rehabilitation medicine, Skåne University Hospital, Malmö, Sweden; Fraunhofer Research Institution of Marine Biotechnology, GERMANY

## Abstract

**Background and purpose:**

Stroke affects mortality, functional ability, quality of life and incurs costs. The primary objective of this study was to estimate the costs of stroke care in Sweden by level of disability and stroke type (ischemic (IS) or hemorrhagic stroke (ICH)).

**Method:**

Resource use during first and second year following a stroke was estimated based on a research database containing linked data from several registries. Costs were estimated for the acute and post-acute management of stroke, including direct (health care consumption and municipal services) and indirect (productivity losses) costs. Resources and costs were estimated per stroke type and functional disability categorised by Modified Rankin Scale (mRS).

**Results:**

The results indicated that the average costs per patient following a stroke were 350,000SEK/€37,000–480,000SEK/€50,000, dependent on stroke type and whether it was the first or second year post stroke. Large variations were identified between different subgroups of functional disability and stroke type, ranging from annual costs of 100,000SEK/€10,000–1,100,000SEK/€120,000 per patient, with higher costs for patients with ICH compared to IS and increasing costs with more severe functional disability.

**Conclusion:**

Functional outcome is a major determinant on costs of stroke care. The stroke type associated with worse outcome (ICH) was also consistently associated to higher costs. Measures to improve function are not only important to individual patients and their family but may also decrease the societal burden of stroke.

## Introduction

Stroke is caused by ischemia (ischemic stroke, IS), hemorrhage (intracerebral hemorrhage, ICH) in the brain or subarachnoid hemorrhage (SAH) in the brain. Early acute treatment, which is vital for survival and minimizing brain damage due to the stroke, has improved for IS with development of reperfusion therapy but no curative therapy is yet available for ICH. Still, many patients have remaining disabilities after a stroke, with life-long consequences on functional ability [1] and quality of life [2, 3]. In Sweden, approximately 25,000 patients suffer from an IS or ICH each year [4] and the effects on morbidity, mortality and costs are substantial [1, 5–8].

Costs and resources associated to stroke care and health outcomes have previously been studied based on Swedish registry data [3, 8–10]. The difference in resource use and costs between stroke types and level of remaining disability is, however, less explored. As treatment alternatives, expected outcome and resource use for patients with ICH or IS differ, it is important to separate the two stroke types in assessments of the costs of stroke. There is also a need to understand differences in resource use related to the actual outcome (e.g. functional disability) after stroke in order to allocate resources efficiently.

Few studies have captured the wide range of resource dimensions associated with the care of stroke patients. Firstly, in Sweden these include county council services, which are divided into specialty care (both inpatient and outpatient services at hospitals) and primary care (outpatient services in primary care centers). Specialty care manages the acute care of stroke patients and specialized rehabilitation. Primary care centers manage primary and secondary prevention as well as long-term rehabilitation services. Secondly, municipalities are responsible for home-based services such as home care service and special housing as well as health care services provided in the home (in some regions a shared responsibility with primary care centers). Thirdly, the state is responsible for social insurance (including sick leave and disability pension) as well as reimbursed pharmaceuticals through grants to the county councils.

As new acute stroke therapies have become available within the last decade, with an emphasis on the acute management of the patients, there has been a shift in prognosis of health outcomes [11–13] and thereby also in resource use [14–17]. This has increased the need for health economic assessments of new therapies, but Swedish data for modelling purposes have been scarce.

The primary objective of this study was to estimate the costs of stroke care by level of disability and stroke type.

## Methods

### Study population and data sources

The research database consisted of patient-level data from patients suffering from stroke 2007–2012, identified in patient administrative systems (PAS) from seven Swedish regions/county councils (Jämtland-Härjedalen, Östergötland, Dalarna, Uppsala, Skåne, Stockholm, and Västra Götaland), covering approximately 60% of all registered strokes annually in Sweden. PAS contain information on diagnosis and procedures related to all health care activities registered within the region (both specialty and primary care). Adult patients (≥18 years) diagnosed with stroke were identified at inpatient admission (ICD-10: I61* (ICH) and I63* (IS)). Although the stroke panorama also can include SAH, at the time, it was not part of the Swedish Stroke Register quality data base. Therefore, the impact of SAH could not be assessed. SAH might also yield a diffuse brain injury in contrast to the focal consequences of the other stroke types and therefore could have a different recovery trajectory. All resource consumption data from the study period was retrieved.

Through the unique personal identification numbers, data was linked to multiple other data sources on individual level: the Swedish Stroke Register (patient characteristics and level of disability), Statistics Sweden (mortality), the National Board of Health and Welfare (municipality services and pharmaceuticals) and the Swedish Social Insurance Agency (sick leave and disability pension). The national coverage of these registries ranges from 95–100%.

The final study population consisted of 47,807 patients diagnosed with stroke (IS or ICH) during 2007–2010, allowing for two years follow-up. Patients not residing in any of the included county councils were excluded from the analysis to allow for complete follow-up.

### Study variables

Identification of resource components associated with stroke care was based on available literature and expert opinion. The resource components were selected to capture as many cost components as possible after stroke, see Table 1. All resource components of county council resources, municipality care and work absence were measured in total values. Home care visits by medical personnel (either by county council or municipality) were excluded due to limited data availability, and home care services were limited to maximum 8,760 hours per year (corresponding to 24-hour service).

**Table 1:** Unit costs.

Resource component	Unit Cost (2016)		Source
	SEK	€	
Inpatient day first year ICH (day)	8,214	867	KPP database
Inpatient day first year IS (day)	7,010	740	KPP database
Inpatient day second year (day)	6,739	712	KPP database
Outpatient visit Speciality care	2,720	287	KPP database
Outpatient visit Primary care	1,124	119	Regional price lists.
Home care service (hour)	440	46	KPB data
Special housing (per day)	1,749	185	KPB data
Work absence (day)	1,357	143	SCB, Skatteverket

Approximately 50% of the patients had filled a prescription for anticoagulants the year following stroke (data on file), the major part of these being vitamin K antagonists (B01AA). This pharmaceutical treatment amounts to an average cost per patient of 500SEK per year (assuming generic substance, 3 tablets/day) and was omitted from the detailed analysis as it would have no considerable impact on the results. However, visits to primary care required for dosage control were included in the resource estimates of primary care visits. As new oral anticoagulants (NOAC) generally are cost neutral to vitamin K antagonists (drug plus visits for dosage controls), the cost estimates for total stroke cost will still be valid at an increase in use of NOAKs if replacing use of vitamin K antagonists.

Resource use was assessed stratified by functional disability using Modified Rankin Scale (mRS). mRS is a commonly used scale to assess the patient’s functional disability after stroke by rating the patient’s disability on a scale from 0 to 6 where:

0 = No symptoms at all

1 = No significant disability despite symptoms; able to carry out all usual duties and activities

2 = Slight disability; unable to carry out all previous activities, but able to look after own affairs without assistance

3 = Moderate disability; requiring some help, but able to walk without assistance

4 = Moderately severe disability; unable to walk without assistance and unable to attend to own bodily needs without assistance

5 = Severe disability; bedridden, incontinent and requiring constant nursing care and attention

6 = Dead.

Categories 0–2 are usually considered good functional outcome [18]. mRS was estimated based on data from the Swedish Stroke Register and in accordance with algorithms developed by Eriksson et al [19]. mRS was assessed at three and twelve months post stroke. The results at three months were used for categorizing first-year resource use and costs, and the twelve months results for second-year estimates.

Patients with good functional outcome can be used as reference case, enabling a valid assessment of the increased resource use by worse functional ability. This data can hence also be used for modelling purposes.

### Costs

Costs were assessed using a societal perspective, i.e. both direct and indirect costs were estimated. Direct costs were summed independent of payer (county council, municipality, state). Indirect costs were estimated based on days of absence from work, defined as either on sick leave or disability pension, estimated using a human capital approach [20].

The unit costs of inpatient stay and outpatient visits in specialist care were retrieved from the cost-per-patient (KPP) database at The Swedish Federation of County Councils (SKL) [21]. First year costs of inpatient care (per day) were identified for patients with main diagnosis ICD-10 I61* and I63* for ICH and IS respectively. By using KPP data, the total cost including the cost of procedures are included in the estimates. Unit costs of inpatient care during second year as well as unit costs for outpatient visits both years were estimated by an unweighted average of daily cost in a general ward, acute care, palliative care, internal medicine, stroke care, geriatric care and rehabilitation. Primary care visit cost was estimated by an average of available regional price lists, using rates for medical doctors and other health care professionals respectively. Home care services and cost for special housing were retrieved from the cost-per-user (KPB) database at SKL [22]. An unweighted average cost of the reporting municipalities was used. Work absence was estimated based on average monthly wage of all sectors, published by SCB, plus employer taxes.

Costs are presented as SEK 2016 and for main results also in Euros, transformed with the annual average exchange rate for 2016 of 9.4707 SEK/€ as reported by the Swedish central bank (Riksbanken).

### Sensitivity analyses

Total amount of resources were used instead of only stroke-related resources as the latter may be subject to bias due to differences in registration of diagnosis codes and the main analyses allows for comparisons within the stroke population. Another possibility would have been to use the patients as their own controls. A sensitivity analysis was therefore performed on the level of costs associated to county council resources prior to stroke. This was done on the population with a stroke in 2009 and 2010 (only surviving patients).

Age could potentially be a strong determinant of costs and functional disability. To assess the impact on the results of age, a stratified analysis was performed of age and level of functional disability for each stroke type and year post stroke.

## Results

### Patient characteristics

The patients with ICH constituted 12% of the total stroke population (Table 2). Compared to patients with IS, they were on average younger at the time of stroke and a slightly larger proportion of the ICH patients were living at home with no home care services. Approximately half of all patients were female and 11% were ADL-dependent (Activities of Daily Living) prior to stroke.

**Table 2:** Descriptive statistics of study sample.

Variable	Intracerebral haemorrhage (ICH)	Ischemic stroke (IS)
Sample size (stroke population)	5,693	42,114
Age (mean)	73.3	76.6
% below 65 years	26.3%	17.9%
Male (%)	53.6%	49.9%
Living arrangements prior to stroke (distribution %)		
- Living at home, no home care service	74.2%	72.1%
- Living at home, with home care service	16.2%	18.7%
- Special housing	9.6%	9.1%
ADL-dependent prior to stroke* (%)	11.0%	11.1%
Previous stroke	21.9%	23.5%

*Defined as requiring help getting dressed and/or going to the toilet.

**Fig 1:**
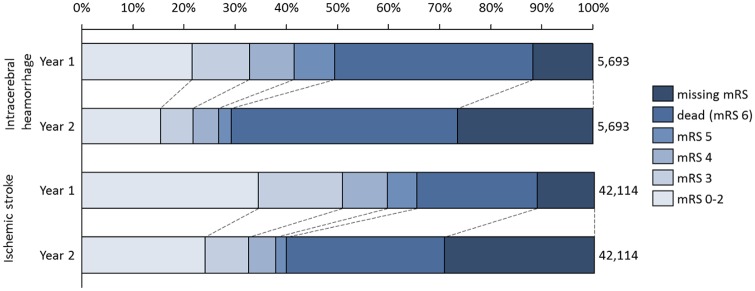
Distribution over mRS categories and missing mRS assessment during first and second year, respectively.

The survival rates at one and two years post stroke were lower for patients with ICH (61% and 56% respectively) compared to patients with IS (77% and 69% respectively) (Fig 1). The division by mRS categories indicate that a higher proportion of patients with IS had a better health outcome (i.e. mRS 0–2) at the first-year assessment (based on mRS at three months), but the proportion decreased by the second-year assessment (based on mRS at twelve months), irrespective of stroke type. However, for second-year assessment, there was a high proportion of patients with missing data on mRS. For this reason, the results of resource use for all surviving patients were also presented.

### Resource use

Patients with ICH consumed more county council resources than patients with IS for each level of functional disability (Table 3), but similar levels of municipality care during the first year after stroke. The results also indicate that resource use increased with higher levels of functional disability, regarding inpatient care and days in special housing, whereas home care services were mostly used by patients with mRS 4, regardless of stroke type. For outpatient care, patients with mRS 5 utilized the least resources.

**Table 3:** Resource use by functional disability and stroke type.

		Haemorrhagic stroke						Ischemic stroke					
		Inpatient stay (days)	Outpatient Speciality care (visits)	Outpatient primary care (visits)	Home care service (hours)	Special housing (days)	Work absence (days)	Inpatient stay (days)	Outpatient Speciality care (visits)	Outpatient primary care (visits)	Home care service (hours)	Special housing (days)	Work absence (days)
First year	mRS (at month 3)												
	0–2	23	11	13	19	2	99	12	9	13	13	1	50
	3	37	10	11	235	34	53	25	8	12	243	34	28
	4	49	12	12	510	82	56	35	9	13	547	75	30
	5	64	6	9	501	170	51	41	5	8	392	213	16
	Dead (1st year)	14	1	1	47	26	4	23	2	3	100	48	3
	All survivors	39	10	12	213	51	80	22	8	12	171	40	41
	All patients	29	7	7	146	41	51	22	7	10	154	42	32
Second year	mRS (at month 12)												
	0–2	2	4	5	25	1	57	3	3	5	26	1	33
	3	6	4	6	698	39	55	6	3	5	571	40	21
	4	7	3	6	1,419	67	58	8	3	5	1,325	78	29
	5	3	2	5	689	250	27	4	3	5	741	265	7
	Dead (2nd year)	12	3	4	453	128	11	14	3	5	505	105	6
	All survivors	5	3	5	429	52	65	5	3	5	373	44	33
	All patients	5	3	5	431	59	60	6	3	5	384	50	31

Note: Values presented are means for total resource use during the first and second year after a stroke, respectively. mRS = modified Rankin Scale.

The highest level of work absence was identified for patients with mRS 0–2. Noteworthy, the proportion of patients under 65 was also highest in this group. When only assessing work absence in the population below 65, the number of days was on average 145 for patients with ICH and 95 for IS, respectively. There was no clear relationship between work absence and level of functional disability in this subpopulation (data on file).

The results for the second year follow a similar pattern when analyzed by functional disability, however at a different level. Patients had lower average level of resource consumption of inpatient and outpatient care but higher levels of municipality care (home care service and special housing) compared to the first-year assessment. No large differences in resource use were identified between stroke types, except higher number of days of work absence for ICH patients (also a larger population under 65).

### Costs

The total per-patient cost during first year for all patients was approximately 470,000SEK/€50,000 and 370,000SEK/€39,000 for patients with ICH and IS respectively. The corresponding costs for the second year were 420,000SEK/€45,000 and 350,000SEK/€37,000. Indirect costs constituted 12–20% of the total costs dependent on year and stroke type (all cost estimates presented in S1 and S2 Tables).

**Fig 2:**
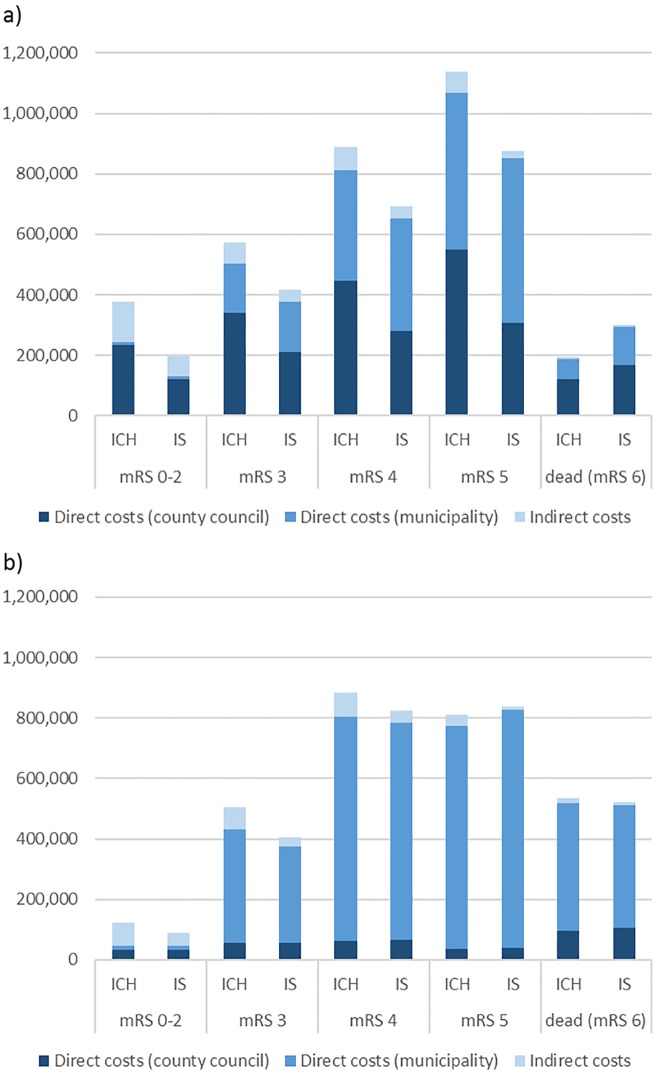
First (a) and second (b) year cost by functional ability and stroke type, per patient.

Fig 2 illustrates the first-year and second-year cost per patient for patients with ICH and IS separated by functional disability. During the first year, costs increased with each level of functional disability, and patients with ICH were associated with higher total average costs for any level of functional disability (apart from dead) compared to patients with IS. These cost differences were primarily driven by the differences in use of inpatient care and special housing. The total average cost ranged from 200,000SEK/€21,000 to 1,100,000SEK/€120,000 for the first year following stroke for the different patient subgroups. mRS 5 was associated with an almost four-fold increase in costs compared to mRS 0–2 for both patients with ICH and IS.

The results further indicate that there were remaining costs for stroke patients in the second year after stroke, especially for patients with continuous functional disability (mRS 4 and mRS 5). ICH was still associated with higher costs for most groups as well as in total, although with a smaller difference to IS than first-year costs. The cost increase of mRS 4 or 5 compared to mRS 0–2 in the second year was approximately eight-fold.

**Fig 3:**
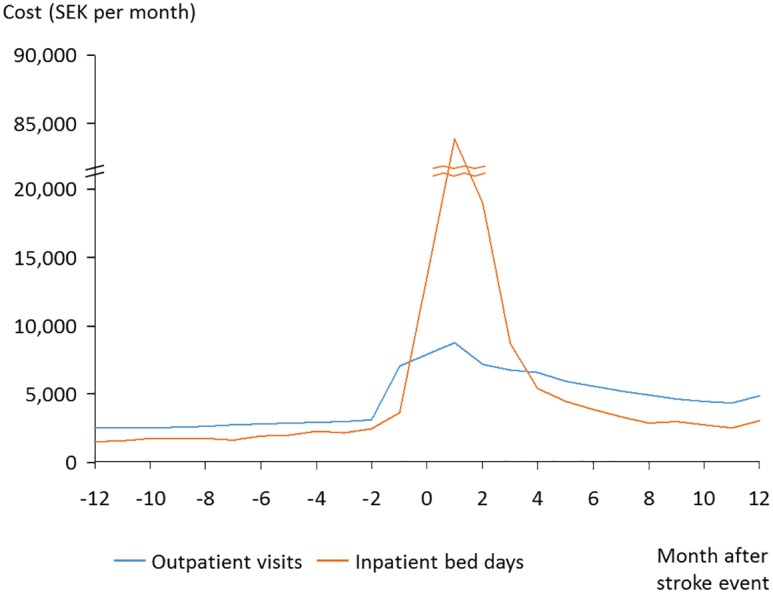
Resource use prior to and after stroke (survivors).

Data indicated that the costs increased in the months prior to the stroke (Fig 3). The three months after stroke was the period with the highest costs, after which the costs decreased. For patients surviving two years, the excess county council costs during the first year after stroke compared to the year prior to stroke was 150,000SEK/€16,000, which constitute approximately 73%. The costs during second year following stroke were 20% higher than the year prior to stroke.

**Fig 4:**
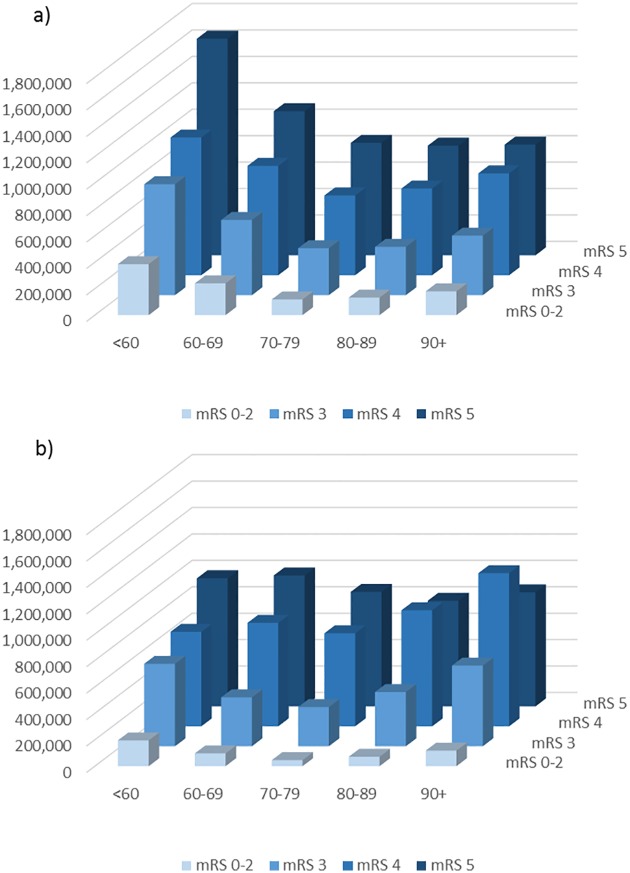
Total cost (SEK) by functional disability and age during first (a) and second (b) year post ischemic stroke.

Stratified analyses by age indicated that for any given age category, there was an increase in total costs for each level of functional disability during the first and second year, although being in the worst state of functional disability (mRS 5) was associated with slightly lower costs than mRS 4 during second year for older age groups (see Fig 4a and 4b for IS patients as illustration, cost estimates in S3 and S4 Tables). Further, the youngest age group was associated with the highest cost, irrespective of functional disability during the initial year. This was primarily driven by higher levels of indirect costs and inpatient stay at worse levels of functional disability.

## Discussion

Functional ability is a strong determinant of cost of stroke in the years following the stroke event. The results from this study indicate higher costs with worse functional disability, up to an eight-fold increase in average per-patient costs during the second year after stroke (comparing mRS 5 to mRS 0–2). These conclusions hold even after stratifying the results by different age groups. This sensitivity analysis further indicated that the youngest population (<60) incurred the highest costs in any level of disability. Previous studies have indicated a similar relationship between functional disability and costs, although estimating costs at a shorter term [23, 24]. This could be of particular interest when estimating cost-benefit of acute treatments such as thrombectomy which has high initial costs but potentially large effects on outcome. If a treatment can shift a patient’s functional outcome from mRS 4–5 to mRS 0–2, the potential benefit could be substantial also in monetary values. It also emphasizes the importance of continuous rehabilitation to maintain functional ability during the years following stroke.

The results indicate that the average costs per patient following stroke were extensive, adding up to 350,000SEK/€37,000–480,000SEK/€50,000 annually for the population at large, but with large variation between different mRS categories, ranging from annual costs of 100,000SEK/€10,000–1,100,000SEK/€120,000 per patient. Costs were higher than previous Swedish estimates [8, 9], probably partly explained by availability of more cost components and inclusion of total costs in this study, compared to previously published findings. Further, the results from this study indicate that indirect costs constituted a minor part of the total costs. The estimates of indirect costs were in line with previous studies [3, 10], but have been estimated to constitute a higher proportion [25]. Patients with ICH consumed more county council resources than patients with IS and were thus associated with higher costs. The difference in costs between stroke types was larger during the first year than the second year following the stroke. Main cost drivers during the first year were county council resources, primarily inpatient care, whereas the second-year cost drivers were primarily municipality resources, dominated by home care services for mRS 0–4 and special housing for patients with mRS 5. These results were demonstrated irrespective of stroke type and in line with previous findings [10]. Notably, as the use of reperfusion therapies increases over time, these costs will add to the total cost during the first year after stroke.

The county council resources during the second year were 30% higher than pre-stroke and some of these costs are likely driven by co-morbidities. However, the main cost drivers during the second year were continuous municipality care and not county council health care activities. Patients with worse functional disability (thereby higher resource use) were to a higher degree ADL-dependent and utilizing municipality care (either home care service or special housing) prior to the stroke, compared to patients with better outcomes. Therefore, the excess costs of the stroke are likely smaller than estimated within this study also for municipality care. However, ADL-dependent patients constituted 11% of the total population, 20% of the patients in mRS 5 the first year and 25% of patients in mRS 5 during the second year following stroke. In addition, analyses have indicated that the vast majority of patients in special housing after stroke were living at home prior to the stroke (data on file). The majority of patients with high levels of consumed municipality resources after stroke were hence expected to have these costs as a consequence of the stroke.

A major strength of this study is its base on registry data with good coverage of the stroke population, minimizing the risk of selection bias. Firstly, the PAS databases cover all patients with acute stroke diagnosed and registered within each county council and all their health care contacts. The included county councils also covered a large part of the Swedish population. Still, generalizations with regards to other regions should be made with caution. Secondly, the study population consisted of patients with stroke diagnosis registered in both PAS and the Swedish Stroke Register (coverage rate >95% [1]), implying that only patients with confirmed stroke diagnosis were included.

Another strength is the study’s combination of several data sources, enabling analyses to include numerous relevant resource components and cost estimates on patient level. The holistic approach used in this study has highlighted the importance of taking all costs into account irrespective of payer (county council, municipality or state). The results indicate that county council resources account for the largest part during the first year, but thereafter municipality resource use constitute the majority of the costs. To enable efficient utilization of societal resources, it is vital that speciality care, primary care and municipality care work closely together as they all have important parts in the prevention and management of stroke patients and are all three associated with substantial costs.

A registry-based study is, however, associated with certain limitations that need to be considered when interpreting the results, such as missing data, incomplete data and incorrect registration. The study population was limited to the patients covered within the Swedish Stroke Register, and therefore patients with SAH were excluded. The cost estimates in this study were conservative as not all cost components associated to stroke could be included due to limitations of data availability. As this study was based on available registry data, information on pre-hospital care, home care visits by medical personnel (by municipalities or county councils) and informal care were not included. Mortality costs were also omitted. Swedish assessments have indicated that informal care and mortality costs constitute 6% of the total costs [10].

Resource use was measured in total amounts of resources utilized after stroke, i.e. not adjusted for pre-stroke levels or only those directly referable to the stroke event. There were also difficulties in determining with precision what resources were directly attributable to the stroke event, due to uncertainties of diagnosis codes, effect of comorbidities etc. An early assessment of diagnosis codes for inpatient stay and outpatient visits in specialty care however showed that approximately 95% of these resources were deemed to be stroke-related the year after stroke (data on file). Alternatively, patients could have acted as their own controls to adjust for pre-stroke resource levels. Results from the sensitivity analysis indicate that resource use of county council resources increased the year prior to the stroke, which may be related to the upcoming stroke event which may underestimate the excess cost of stroke if compared to the year before. Still, in such an analysis, 73% of the costs could be considered excess costs the year after compared to the year before, which is lower than the 95% estimated based on diagnosis codes. To enable comparisons of cost differences, resource use was instead assessed based on functional disability assessed by the Modified Rankin Scale (mRS), allowing to use mRS 0–2 (good functional outcome) as reference population. The results, based on functional disability in this study, may provide deeper insights when assessing health care interventions post stroke (in e.g. health economic modelling). However, for assessments of preventive interventions a comparison needs to be made to the pre-stroke resources and costs, potentially adjusting for the rising costs just prior to the stroke event.

Categorization based on estimated mRS was available at three and twelve months after stroke. Second-year costs were therefore based on mRS at one year, assuming that patients remain in this stage the whole year except in the case of death. However, patients may be at a worse state during the second year (due to aging and accelerating co-morbidities) whereby the estimates for mRS 4 especially may be overestimated.

The costs following a stroke were extensive during the first year and beyond. The results indicate increasing costs with worse functional disability, and higher costs for patients with ICH compared to IS during the first year after stroke. The results from this study emphasize the importance of fast and efficient management of stroke patients to decrease the likelihood of permanent functional disability. Also, interventions such as thrombectomy that may dramatically improve outcome for IS can be cost-effective even if the initial cost is high due to the long-term costs associated with worse functional disability. The need of continuous rehabilitation to maintain functional ability is obvious. This is not only important for the individual patients, reaching better health outcomes, but also for society at large, in decreasing the societal burden of stroke by keeping patients in better health states associated with lower costs.

## Supporting information

S1 Table Cost hemorrhagic stroke (SEK and Euro). (DOCX)

S2 Table Costs ischemic stroke (SEK and Euro). (DOCX)

S3 Table Total cost by age category and level of functional disability for ICH during first and second year post stroke, respectively (SEK and Euro). (DOCX)

S4 Table Total cost by age category and level of functional disability for IS during first and second year post stroke, respectively (SEK and Euro). (DOCX)
