# Supplementary material for: Relationship between functional disability and costs one and two years post stroke
Source: PLoS One. 2017 Apr 6;12(4):e0174861. doi: 10.1371/journal.pone.0174861 (PMC5383241; doi:10.1371/journal.pone.0174861)
Supplement: S1 Table — (DOCX) [file pone.0174861.s001.docx]

Supporting Information

**S1 Table. Cost hemorrhagic stroke (SEK and Euro)**

|  | | Inpatient stay | | Outpatient Speciality care | | Outpatient primary care | | Home care service | | Special housing | | Work absence | |
| --- | --- | --- | --- | --- | --- | --- | --- | --- | --- | --- | --- | --- | --- |
|  |  | SEK | Euro | SEK | Euro | SEK | Euro | SEK | Euro | SEK | Euro | SEK | Euro |
| First year | mRS 0-2 | 189,558 | 20,015 | 29,575 | 3,123 | 14,442 | 1,525 | 8,298 | 876 | 3,124 | 330 | 133,681 | 14,115 |
|  | mRS 3 | 299,920 | 31,668 | 26,634 | 2,812 | 12,409 | 1,310 | 103,265 | 10,904 | 59,570 | 6,290 | 71,371 | 7,536 |
|  | mRS 4 | 400,566 | 42,295 | 31,806 | 3,358 | 13,011 | 1,374 | 224,302 | 23,684 | 142,728 | 15,071 | 76,489 | 8,076 |
|  | mRS 5 | 524,393 | 55,370 | 15,200 | 1,605 | 10,126 | 1,069 | 220,328 | 23,264 | 298,100 | 31,476 | 69,420 | 7,330 |
|  | Dead (1 yr) | 117,408 | 12,397 | 3,097 | 327 | 1,205 | 127 | 20,622 | 2,177 | 45,269 | 4,780 | 5,315 | 561 |
|  | All survivers | 317,463 | 33,521 | 27,447 | 2,898 | 12,970 | 1,369 | 93,505 | 9,873 | 89,913 | 9,494 | 108,765 | 11,484 |
|  | All patients | 239,662 | 25,306 | 17,977 | 1,898 | 8,394 | 886 | 64,104 | 6,769 | 72,551 | 7,661 | 68,534 | 7,236 |
| Second year | mRS 0-2 | 16,134 | 1,704 | 9,784 | 1,033 | 5,927 | 626 | 10,943 | 1,155 | 2,181 | 230 | 77,797 | 8,215 |
|  | mRS 3 | 40,378 | 4,263 | 9,770 | 1,032 | 6,313 | 667 | 307,039 | 32,420 | 67,385 | 7,115 | 75,023 | 7,922 |
|  | mRS 4 | 46,398 | 4,899 | 9,032 | 954 | 6,388 | 674 | 624,411 | 65,931 | 117,890 | 12,448 | 78,650 | 8,305 |
|  | mRS 5 | 23,346 | 2,465 | 6,217 | 656 | 5,211 | 550 | 303,053 | 31,999 | 437,750 | 46,221 | 36,352 | 3,838 |
|  | Dead (2 yr) | 82,740 | 8,736 | 7,431 | 785 | 4,992 | 527 | 199,412 | 21,056 | 223,226 | 23,570 | 15,271 | 1,612 |
|  | All survivers | 30,894 | 3,262 | 8,159 | 862 | 5,567 | 588 | 188,760 | 19,931 | 90,738 | 9,581 | 88,153 | 9,308 |
|  | All patients | 35,454 | 3,744 | 8,095 | 855 | 5,516 | 582 | 189,614 | 20,021 | 102,391 | 10,811 | 81,742 | 8,631 |

Note: mRS for first year is estimatated at 3 months, mRS for second year is estimated at 12 months
