# Supplementary material for: Relationship between functional disability and costs one and two years post stroke
Source: PLoS One. 2017 Apr 6;12(4):e0174861. doi: 10.1371/journal.pone.0174861 (PMC5383241; doi:10.1371/journal.pone.0174861)
Supplement: S2 Table — (DOCX) [file pone.0174861.s002.docx]

Supporting Information

**S2 Table. Costs ischemic stroke (SEK and Euro)**

|  | | Inpatient stay | | Outpatient Speciality care | | Outpatient primary care | | Home care service | | Special housing | | Work absence | |
| --- | --- | --- | --- | --- | --- | --- | --- | --- | --- | --- | --- | --- | --- |
|  |  | SEK | Euro | SEK | Euro | SEK | Euro | SEK | Euro | SEK | Euro | SEK | Euro |
| First year | mRS 0-2 | 83,833 | 8,852 | 24,061 | 2,541 | 14,547 | 1,536 | 5,798 | 612 | 2,327 | 246 | 68,164 | 7,197 |
|  | mRS 3 | 176,040 | 18,588 | 22,457 | 2,371 | 13,695 | 1,446 | 106,885 | 11,286 | 59,569 | 6,290 | 38,003 | 4,013 |
|  | mRS 4 | 242,243 | 25,578 | 23,598 | 2,492 | 14,210 | 1,500 | 240,716 | 25,417 | 131,086 | 13,841 | 40,293 | 4,255 |
|  | mRS 5 | 286,535 | 30,255 | 12,535 | 1,324 | 9,035 | 954 | 172,452 | 18,209 | 373,093 | 39,394 | 21,105 | 2,229 |
|  | Dead (1 yr) | 159,715 | 16,864 | 5,218 | 551 | 2,865 | 303 | 44,138 | 4,661 | 83,313 | 8,797 | 4,084 | 431 |
|  | All survivers | 151,998 | 16,049 | 22,604 | 2,387 | 13,631 | 1,439 | 75,325 | 7,953 | 69,903 | 7,381 | 56,242 | 5,939 |
|  | All patients | 153,799 | 16,239 | 18,545 | 1,958 | 11,118 | 1,174 | 67,965 | 7,176 | 72,506 | 7,656 | 44,066 | 4,653 |
| Second year | mRS 0-2 | 18,186 | 1,920 | 8,604 | 908 | 5,616 | 593 | 11,658 | 1,231 | 2,268 | 239 | 44,326 | 4,680 |
|  | mRS 3 | 39,930 | 4,216 | 9,158 | 967 | 6,146 | 649 | 251,063 | 26,509 | 69,169 | 7,303 | 28,149 | 2,972 |
|  | mRS 4 | 50,549 | 5,337 | 8,420 | 889 | 5,406 | 571 | 583,200 | 61,579 | 137,045 | 14,470 | 39,769 | 4,199 |
|  | mRS 5 | 24,820 | 2,621 | 8,840 | 933 | 5,640 | 596 | 325,881 | 34,409 | 463,114 | 48,900 | 9,016 | 952 |
|  | Dead (2 yr) | 91,986 | 9,713 | 9,039 | 954 | 5,683 | 600 | 222,324 | 23,475 | 183,852 | 19,413 | 8,157 | 861 |
|  | All survivers | 31,527 | 3,329 | 8,710 | 920 | 5,733 | 605 | 164,128 | 17,330 | 76,341 | 8,061 | 44,981 | 4,749 |
|  | All patients | 37,392 | 3,948 | 8,710 | 920 | 5,728 | 605 | 169,118 | 17,857 | 86,771 | 9,162 | 41,409 | 4,372 |

Note: mRS for first year is estimatated at 3 months, mRS for second year is estimated at 12 months
