# Supplementary material for: Relationship between functional disability and costs one and two years post stroke
Source: PLoS One. 2017 Apr 6;12(4):e0174861. doi: 10.1371/journal.pone.0174861 (PMC5383241; doi:10.1371/journal.pone.0174861)
Supplement: S3 Table — (DOCX) [file pone.0174861.s003.docx]

Supporting Information

**S3 Table. Total cost by age category and level of functional disability for ICH during first and second year post stroke, respectively (SEK and Euro)**

|  | Age category | <60 | | 60-69 | | 70-79 | | 80-89 | | 90+ | |
| --- | --- | --- | --- | --- | --- | --- | --- | --- | --- | --- | --- |
|  |  | SEK | Euro | SEK | Euro | SEK | Euro | SEK | Euro | SEK | Euro |
| First year total costs | Functional disability at 3 monts | | | | | | | | | | |
|  | mRS 0-2 | 585,491 | 61,821 | 402,176 | 42,465 | 201,222 | 21,247 | 192,124 | 20,286 | 520,131 | 54,920 |
|  | mRS 3 | 1,118,617 | 118,113 | 746,643 | 78,837 | 447,938 | 47,297 | 431,574 | 45,569 | 495,130 | 52,280 |
|  | mRS 4 | 1,511,305 | 159,577 | 1,037,867 | 109,587 | 716,278 | 75,631 | 766,103 | 80,892 | 865,887 | 91,428 |
|  | mRS 5 | 2,291,237 | 241,929 | 1,441,171 | 152,171 | 924,660 | 97,634 | 911,833 | 96,279 | 1,054,997 | 111,396 |
| Second year total costs | Functional disability at 1 year | | | | | | | | | | |
|  | mRS 0-2 | 235,001 | 24,813 | 96,460 | 10,185 | 39,674 | 4,189 | 107,011 | 11,299 | 191,674 | 20,239 |
|  | mRS 3 | 755,793 | 79,803 | 410,961 | 43,393 | 446,047 | 47,098 | 527,907 | 55,741 | 505,665 | 53,393 |
|  | mRS 4 | 819,131 | 86,491 | 700,723 | 73,988 | 921,706 | 97,322 | 993,686 | 104,922 | 1,144,388 | 120,835 |
|  | mRS 5 | 778,243 | 82,174 | 648,595 | 68,484 | 658,951 | 69,578 | 979,117 | 103,384 | 839,824 | 88,676 |
