# Supplementary material for: Relationship between functional disability and costs one and two years post stroke
Source: PLoS One. 2017 Apr 6;12(4):e0174861. doi: 10.1371/journal.pone.0174861 (PMC5383241; doi:10.1371/journal.pone.0174861)
Supplement: S4 Table — (DOCX) [file pone.0174861.s004.docx]

Supporting Information

**S4 Table. Total cost by age category and level of functional disability for IS during first and second year post stroke, respectively (SEK and Euro)**

|  | Age category | <60 | | 60-69 | | 70-79 | | 80-89 | | 90+ | |
| --- | --- | --- | --- | --- | --- | --- | --- | --- | --- | --- | --- |
|  |  | SEK | Euro | SEK | Euro | SEK | Euro | SEK | Euro | SEK | Euro |
| First year total costs | Functional disability at 3 monts | | | | | | | | | | |
|  | mRS 0-2 | 385,664 | 40,722 | 240,629 | 25,408 | 118,709 | 12,534 | 132,688 | 14,010 | 180,120 | 19,019 |
|  | mRS 3 | 839,801 | 88,674 | 569,492 | 60,132 | 355,686 | 37,556 | 365,870 | 38,632 | 450,299 | 47,547 |
|  | mRS 4 | 1,042,260 | 110,051 | 827,596 | 87,385 | 603,660 | 63,740 | 656,248 | 69,292 | 771,316 | 81,442 |
|  | mRS 5 | 1,639,477 | 173,110 | 1,090,833 | 115,180 | 850,987 | 89,855 | 829,671 | 87,604 | 837,706 | 88,452 |
| Second year total costs | Functional disability at 1 year | | | | | | | | | | |
|  | mRS 0-2 | 195,271 | 20,618 | 96,676 | 10,208 | 46,278 | 4,886 | 71,393 | 7,538 | 117,774 | 12,436 |
|  | mRS 3 | 624,103 | 65,898 | 370,171 | 39,086 | 296,712 | 31,329 | 410,603 | 43,355 | 610,764 | 64,490 |
|  | mRS 4 | 715,122 | 75,509 | 783,025 | 82,679 | 704,643 | 74,402 | 878,293 | 92,738 | 1,158,913 | 122,368 |
|  | mRS 5 | 969,605 | 102,379 | 991,150 | 104,654 | 866,442 | 91,487 | 799,701 | 84,440 | 863,381 | 91,163 |
